# Characterization Factors to Assess Land Use Impacts on Pollinator Abundance in Life Cycle Assessment

**DOI:** 10.1021/acs.est.2c05311

**Published:** 2023-02-13

**Authors:** Elizabeth M. Alejandre, Laura Scherer, Jeroen B. Guinée, Marcelo A. Aizen, Matthias Albrecht, Mario V. Balzan, Ignasi Bartomeus, Danilo Bevk, Laura A. Burkle, Yann Clough, Lorna J. Cole, Casey M. Delphia, Lynn V. Dicks, Michael P.D. Garratt, David Kleijn, Anikó Kovács-Hostyánszki, Yael Mandelik, Robert J. Paxton, Theodora Petanidou, Simon Potts, Miklós Sárospataki, Catharina J.E. Schulp, Menelaos Stavrinides, Katharina Stein, Jane C. Stout, Hajnalka Szentgyörgyi, Androulla I. Varnava, Ben A. Woodcock, Peter M. van Bodegom

**Affiliations:** † Institute of Environmental Sciences (CML), Leiden University, P.O. Box 9518, 2300 RA Leiden, The Netherlands; ‡ Delft University of Technology, Mekelweg 5, 2628 CD Delft, The Netherlands; § Grupo de Ecología de la Polinización, INIBIOMA, Universidad Nacional del Comahue-CONICET, Quintral 1250, 8400 Bariloche, Río Negro, Argentina; ∥ Agroecology and Environment, Agroscope, Reckenholzstrasse 191, 8046 Zurich, Switzerland; ⊥ Institute of Applied Sciences, Malta College of Arts, Science and Technology (MCAST), PLA9032 Paola, Malta; # Estación Biológica de Doñana (EBD-CSIC)^RINGGOLD^, Avda. Américo Vespucio 26, Isla de la Cartuja, E-41092 Sevilla, Spain; ¶ National Institute of Biology, 1000 Ljubljana, Slovenia; ∇ Department of Ecology, Montana State University, Bozeman, Montana 59717, United States; ○ Centre for Environmental and Climate Science, Lund University, Sölvegatan 37, 22362 Lund Sweden; ⧫ Integrated Land Management, SRUC, JF Niven Building, Auchincruive Estate, KA6 5HW AYR, U.K.; †† Montana Entomology Collection, Montana State University, Room 50 Marsh Laboratory, Bozeman, Montana 59717, United States; ‡‡ Department of Zoology, University of Cambridge, Downing Street, CB2 3EJ Cambridge U.K.; §§ School of Biological Sciences, University of East Anglia, Norwich Research Park, NR4 7TJ Norwich U.K.; ∥∥ University of Reading, RG6 6AR Reading, U.K.; ⊥⊥ Plant Ecology and Nature Conservation Group, Wageningen University & Research, Droevendaalsesteeg 3a, 6708 PB Wageningen, The Netherlands; ## Centre for Ecological Research, Institute of Ecology and Botany, Lendület Ecosystem Services Research Group, Alkotmány str. 2-4, H-2163 Vácrátót, Hungary; ¶¶ Department of Entomology, Faculty of Agriculture Food and Environment, The Hebrew University of Jerusalem, P.O.Box 12, 7610001 Rehovot, Israel; ∇∇ Institute for Biology, Martin Luther University Halle-Wittenberg, Halle-Jena-Leipzig, Hoher Weg 8, 06120 Halle (Saale), Germany; ○○ German Centre for Integrative Biodiversity Research (iDiv), Puschstrasse 4, 04103 Leipzig, Germany; ⧫⧫ Laboratory of Biogeography and Ecology, Department of Geography, University of the Aegean, 81100 Mytilene, Greece; ††† Department of Zoology and Ecology, Institute for Wildlife Management and Nature Conservation, Hungarian University of Agriculture and Life Sciences, Páter K. u. 1., H2100 Gödöllő, Hungary; ‡‡‡ Department of Environmental Geography, Institute for Environmental Studies, Vrije Universiteit Amsterdam, De Boelelaan 1085, 1081 HV Amsterdam, The Netherlands; §§§ Department of Agricultural Sciences, Cyprus University of Technology, Arch. Kyprianos 30, 3036 Lemesos, Cyprus; ∥∥∥ Institute of Biological Sciences, Department of Botany and Botanical Garden, University of Rostock, Wismarsche Strasse 45, 18051 Rostock, Germany; ⊥⊥⊥ Trinity College Dublin, College Green, D02 PN40 Dublin 2, Ireland; ### Department of Plant Ecology, Institute of Botany, Jagiellonian University, ul. Gronostajowa 3, 30-387 Kraków, Poland; ¶¶¶ UK Centre for Ecology & Hydrology, Crowmarsh Gifford, Wallingford, Oxfordshire OX10 8BB, U.K.

**Keywords:** pollinator abundance, ecosystem service, Delphi expert elicitation, agriculture, impact assessment

## Abstract

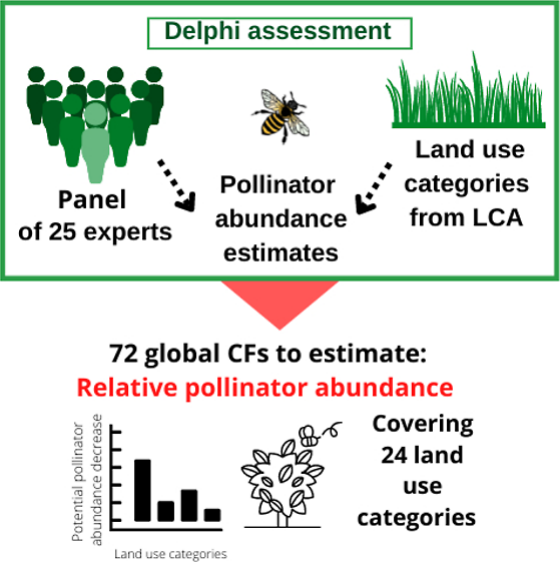

While wild pollinators play a key role in global food production, their assessment is currently missing from the most commonly used environmental impact assessment method, Life Cycle Assessment (LCA). This is mainly due to constraints in data availability and compatibility with LCA inventories. To target this gap, relative pollinator abundance estimates were obtained with the use of a Delphi assessment, during which 25 experts, covering 16 nationalities and 45 countries of expertise, provided scores for low, typical, and high expected abundance associated with 24 land use categories. Based on these estimates, this study presents a set of globally generic characterization factors (CFs) that allows translating land use into relative impacts to wild pollinator abundance. The associated uncertainty of the CFs is presented along with an illustrative case to demonstrate the applicability in LCA studies. The CFs based on estimates that reached consensus during the Delphi assessment are recommended as readily applicable and allow key differences among land use types to be distinguished. The resulting CFs are proposed as the first step for incorporating pollinator impacts in LCA studies, exemplifying the use of expert elicitation methods as a useful tool to fill data gaps that constrain the characterization of key environmental impacts.

## Abstract 2

This study applies a Delphi expert elicitation to derive globally representative characterization factors for estimating land use impacts on the relative wild pollinator abundance for Life Cycle Assessment.

## 1 Introduction

Pollinator communities around the world play a key role in agricultural production by influencing crop quality and yield.^1−5^ Wild pollinators, which provide long-term and effective crop pollination services,^5−7^ have been observed to decline in range and abundance in recent decades.^8−10^ While multiple factors, such as climate change and pesticide use, have been identified as drivers affecting pollinator communities,^11−15^ land use and land management changes remain primary drivers for the decrease in abundance.^16−20^ This decline leads to potential mismatches between the provision of pollination services and the global demand for crop pollination.^9,21−23^ Addressing the potential impact of land use on wild pollinators is therefore essential to help prevent further decline and identify better practices, and it should be incorporated into commonly applied environmental assessment methods used worldwide such as Life Cycle Assessment (LCA).^24,25^

LCA is an internationally standardized (ISO) method used globally to help estimate environmental impacts associated with a product system or service.^26^ The estimation of impacts in LCA studies relies on the translation of inventory flows (which compile information such as resources and emissions) into impacts through the use of characterization factors (CFs; numerical values representing the potential contribution to an environmental impact). Despite the relevance of wild pollinators, their assessment has not been explicitly incorporated in common LCA studies. While recent efforts have provided recommendations for their incorporation in LCA^27,28^ and a characterization model,^29^ LCA studies currently still lack the ability to reflect impacts on pollinator communities since there are no readily applicable CFs that can translate environmental interventions into this specific impact. To address this gap, this study makes use of an expert elicitation assessment, the Delphi method, to obtain estimates of the relative abundance of wild pollinators associated with a variety of land use categories for the production of readily applicable CFs to assess land use impacts.

To guarantee compatibility of the resulting CFs with common LCA inventory flows, this study focuses on the characterization of land use categories found in the widely applied database ecoinvent.^30^ Ecoinvent is one of the largest and most commonly used LCA databases around the world. The database contains information regarding unit process inputs and outputs and provides, in some cases, country-specific information as well as global average values. For this study, the relevant land use categories listed in ecoinvent are characterized to facilitate compatibility and direct application and to encourage the incorporation of a category assessing impacts on pollinators in impact assessment methods, such as ReCiPe2016^31^ and LC-Impact,^32^ among others.^33−35^ We expect the application of the resulting CFs to be a first step toward a more comprehensive assessment of land use impacts on wild pollinators and to illustrate the use of expert elicitation methods as a useful tool to fill gaps where key data might be unavailable for the production of CFs for LCA.

## 2 Methods

### 2.1 Characterization Model for Land Use Impacts on Pollinator Abundance

To produce CFs, we applied a published model that characterizes land use impacts on pollinator abundance in a compatible way with LCA.^29^ The CFs are produced by estimating the difference in pollinator abundance associated with a given land use *x* (PA_*x*_) in reference to the land type that is typically associated with the maximum number of pollinators per m^2^ (PA_ref_). The pollinator density associated with each land category is based on relative expert estimates (*S*_*x*_), which are used to derive the CFs in reference to the most typically abundant land category^29^ as follows
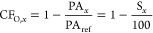


The resulting CFs help translate land use inventory flows (specifically land “occupation” flows as denoted in LCA terminology, in m^2^·year) into relative pollinator abundance impacts. The indicator result, in this case the change in relative pollinator abundance for occupation impacts (RPAO), is calculated by aggregating all occupation flows (O_*x*_) after being multiplied by their respective CFs 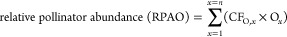
where O_*x*_ is the time-integrated area of occupation in m^2^·year. The unit of the indicator result RPAO is also m^2^·year. The indicator result can be interpreted as the impact on the relative abundance of wild pollinators that is associated with the studied system. In the case of land use change (also referred to in LCA as land transformation), CFs would be derived by estimating the difference in the relative pollinator abundance between two different land use types and multiplying by a regeneration time according to UNEP-SETAC guidelines to obtain compatible units that would allow for aggregation of land use impacts in LCA.^36,37^ However, due to discrepancies in the operationalization of land “transformation” impact assessment,^29,38^ we focus in this study on the derivation of applicable CFs for land “occupation” impacts, referred to simply as land use.

### 2.2 Deriving Pollinator Abundance Estimates (*S*_*x*_)

To derive the pollinator abundance estimates associated with each of the land use types assessed and to determine a reference land use type, we conducted a Delphi assessment (described in detail in Section 2.4). A Delphi assessment is an expert elicitation method that relies on iterative rounds where experts reconsider their scores based on intermediate rounds of feedback and argumentation.^39−41^ For this study, we consulted an international panel of 25 experts, covering 16 nationalities and with expertise across 45 countries (see Supporting Information A, Figure S1). The experts specialize in disciplines relevant to the topic of pollinators and pollination, some with first-hand experience conducting empirical field studies in different land-use types and agricultural crops for different regions of the globe and some with expertise in modeling relationships between land-use and pollinators. All participants remained anonymous to each other during the assessment to encourage equal participation and avoid overpowering dynamics. The assessment was carried out digitally through the Qualtrics survey software (www.qualtrics.com).

**Figure 1:**
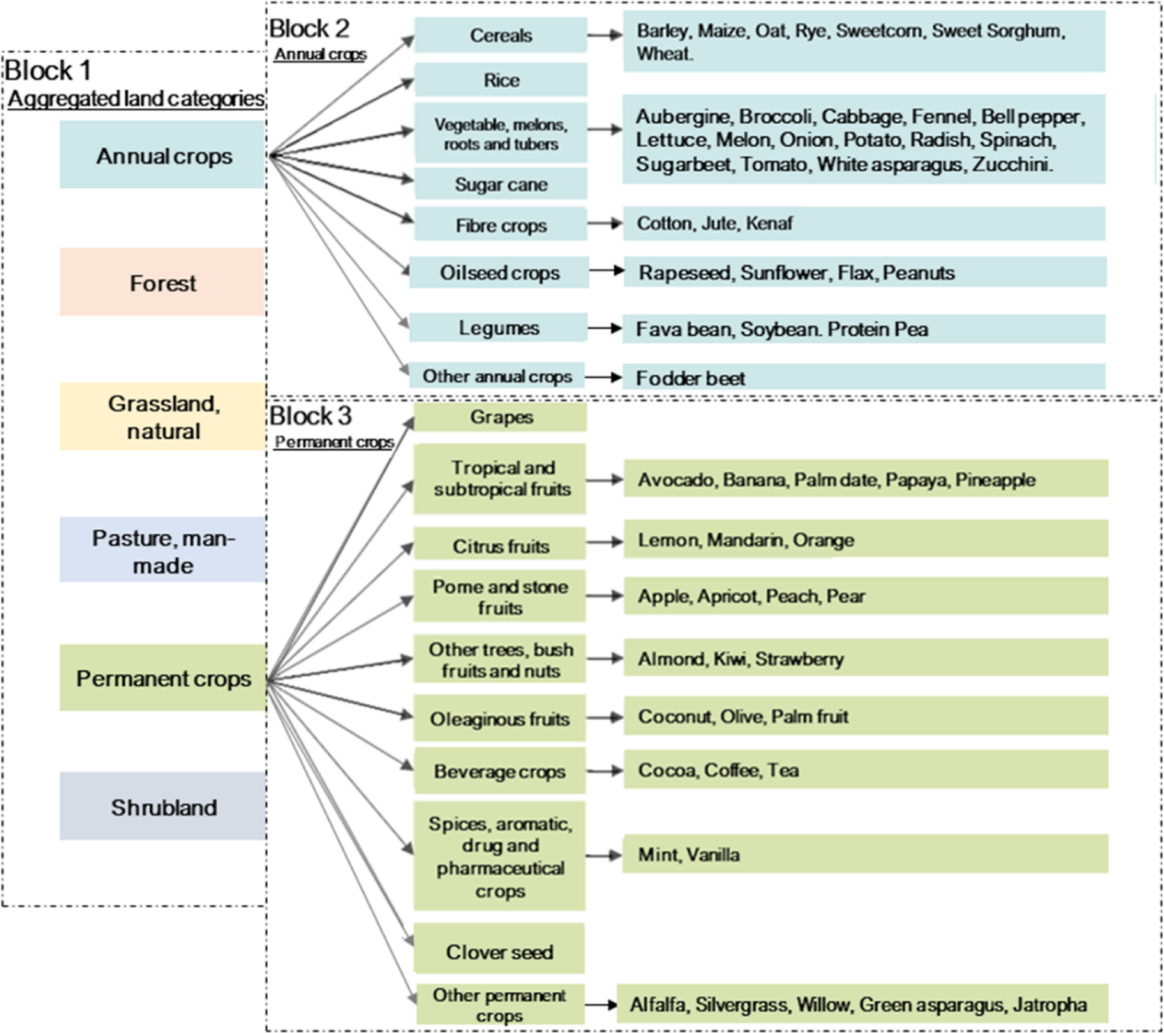
Land use categories assessed for impact characterization.

The participants were asked to provide relative estimates of wild pollinator abundance by considering the foraging characteristics and nesting resources that can be typically associated with the land categories assessed and to consider the potential influence of different land management practices. The relative scores were provided for a series of land use categories that were derived from the ecoinvent database (https://www.ecoinvent.org/) (see Section 2.3). The categories were divided into three blocks (described in detail in Section 2.4). Block 1 consisted of the major aggregated land categories, and blocks 2 and 3 consisted of subgroups for annual and permanent crops, respectively (Figure 1). Examples of the specific crops within each subgroup listed in ecoinvent were provided to the participants in the survey to be taken into consideration for their scores.

Throughout the three rounds of assessment, the feedback provided by experts on their argumentation for pollinator abundance estimates was used for the interpretation of the scores and to help prevent and identify potential misunderstandings that could lead to false outliers. In case scores deviated significantly from the norm, the scores were corroborated with the written justification or direct contact with the expert to verify that the estimates were due to true dissent and not a result of potential misunderstanding. In the latter case, the scores provided by the expert were annulled from the entire round to avoid biases that could have been created by removing single values.

### 2.3 Selection of Land Use Types for Characterization

The land use categories assessed in this study were primarily derived from the ecoinvent life cycle inventory database. These comprise six main categories (grassland, forest, permanent crops, annual crops, pasture, and shrubland) listed for characterization in block 1 (Figure 1). Additional subcategories of annual and permanent crops were assessed in block 2 and block 3 (Figure 1) for characterization and comparison. Crops that were identified by experts as misclassified during the first round of assessment (e.g., rapeseed originally classified as cereal) were corrected and assessed as separate categories during the third round of Delphi.

### 2.4 Delphi Assessment Procedure

Experts were asked to provide pollinator abundance scores from 0 to 100, starting by assigning the maximum value to the category they considered as the reference (the one with the typically highest expected pollinator abundance) and then ranking the rest of the categories accordingly, assessing each block individually. The experts provided world-generic scores for the **typical** pollinator abundance (“typical”” defined as the most expected or representative value, equivalent to the mathematical term “mode”), as well as estimates for the **lowest** and **highest** pollinator abundance that could be associated with each land type by considering not only foraging and nesting resources but also the potential differences due to management practices and biogeographical variations. The participants provided a short, written justification or description of the considerations taken for each score (e.g., habitat characteristics, management practice considered, or trends) and rated their confidence level for the typical estimates on a three-point Likert scale (low, moderate, or high). This estimation of confidence facilitated subsequent discussions by providing a basis of reference for the expertise of otherwise anonymous participants. These confidence scores served in the interpretation and discussion of the results and were not used quantitatively.

At the end of each round, a statistical summary of the results (including mean and range of scores) was shared among the participants, along with an anonymous summary of the argumentations provided by the experts. The participants were asked to consider the argumentations for each category and resubmit their scores. At the end of the second round, the categories that did not reach consensus were submitted for a third and final round of evaluation. The consensus was measured through the coefficient of variation, estimated as the standard deviation (SD) divided by the mean and multiplied by a hundred. A coefficient of variation of ≤50 was considered as a threshold for consensus. The typical, low, and high estimates were treated as independent values. At the end of the third round, the values that did not reach consensus were highlighted as not readily applicable without further evaluation.

### 2.5 Statistical Processing of Delphi Assessment Results

The results of the Delphi assessment were used to derive the relative estimates of pollinator abundance for the calculation of CFs. In block 1, the land category selected by most experts as the one expected to present, on average, the highest typical pollinator abundance, was treated as the reference land category. The typical values attributed by each participant to the reference land type were set to 100, and the rest of the values were scaled accordingly. In blocks 2 and 3, experts provided estimates of abundance from 0 to 100 for subcategories of annual and permanent crops. These values were normalized by setting the maximum typical value provided by each participant as the normalized mean of the high abundance of annual and permanent crops in block 1. For example, if the normalization of block 1 results in a mean high abundance of 40 for annual crops, the maximum typical estimates in block 2 are set to 40, and the rest of the values are scaled. High-abundance estimates can still result in values above 40 after scaling with the reference land. By normalizing blocks 2 and 3 with the high-abundance estimates, a wider range of pollinator abundance can be reflected for the subcategories of annual and permanent crops. This decreases the potential bias from normalizing in reference to, for example, the mean of typical values only or the average across typical, low, and high estimates.

At the end of the Delphi assessment, the resulting normalized *S*_*x*_ estimates were converted to CFs for each land use category, applying the model described in Section 2.1. The mean CFs for typical, low, and high abundance are presented for each land use category, along with their SD, which reflects the between-experts uncertainty of the CF. Additionally, to reflect variations associated with, for example, both biogeographical and management differences, and for cases where it is not known if the typical-, low-, or high-abundance CF would be more appropriate, we combined all the typical, low, and high CFs and calculated the SD, resulting in the combined uncertainty for each land category. Lastly, given that the typical estimates represent, as its name denotes, the most typically expected abundance, we calculated the SD combining all the typical, low, and high CFs, accounting for typical CFs twice, to provide a weighted uncertainty measure for each land use category.

## 3 Results

### 3.1 Pollinator Abundance Estimates

Based on the results of the Delphi assessment, natural grassland was selected by most experts as the reference land type, with shrubland as a close second. The estimates for the other land use types were treated relative to grassland and were scaled accordingly for each of the participants’ estimates as described in Section 2.5. All normalized *S*_*x*_ estimates are provided in Supporting Information B. In block 1, the mean for typical abundance estimates ranged between values of 36 and 100, as shown in Figure S2 (Supporting Information A). Forests, permanent crops, and pastures were rated with intermediate abundance estimates, while annual crops was rated as the land use category presenting typically the lowest abundance. The mean low-abundance estimates varied between 7 and 52 across land categories and mean high estimates between 75 and 120. The largest range observed between the minimum and maximum values for typical and high-abundance estimates in block 1 occurs for the category of forest.

A higher level of land use specificity was assessed in block 2, covering subcategories of annual crops. The estimates of block 2 were normalized in reference to grassland, based on the normalized high mean abundance estimate of 78.6 for annual crops in block 1. The normalized mean of *S*_*x*_ estimates for typical pollinator abundance varies between values of 9 and 76, while the mean of low estimates varies between 1 and 27, and for high boundaries, it varies between 29 and 116 (see Supporting Information A, Figure S3). Sugar cane and rice were rated as crops with a typically low abundance, while the category vegetables, melons, roots, and tubers was rated by most experts as the most likely one to present a higher pollinator abundance, with a mean *S*_*x*_ value of 76. The typical estimate for rice, cereals, and other annual crops did not reach consensus (see Supporting Information A, Figure S4).

In block 3, the subcategory of permanent crops was normalized in reference to grassland, assuming the mean normalized high-abundance value of 93.11 in block 1 as the maximum typical abundance in block 3. The normalized mean estimates for a typical pollinator abundance vary between 30 and 88 across permanent crops, while the values for mean low-abundance estimates range between 8 and 51 and the mean high abundance estimated between 65 and 115 in reference to grassland (Supporting Information A, Figure S5). All estimates for typical and high-abundance rates reached consensus (Supporting Information A, Figure S4), and only five out of ten categories did not reach consensus for low-abundance estimates. The category of pome and stone fruits was rated as the most typically pollinator abundant category from block 3, with a mean normalized value of 87.68.

The initially high divergence observed for the typical abundance estimates for rice and the low-abundance estimates for annual crops, forest, and permanent crops decreased by almost half after three rounds (Supporting Information A, Figure S4). A coefficient of variation of ≤50% was not reached, but the results suggest that additional rounds of scoring and active argumentation could potentially lead to representative and convergent values for these categories. On the other hand, the low-abundance estimates for categories such as cereals, rice, sugar cane, and fiber crops presented a consistently high divergence across all three rounds of scoring, indicating dissent for those crops and/or lesser confidence in the case of rice. Overall, increasing the level of specificity for the aggregated land use categories of annual and permanent crops (moving from block 1 to blocks 2 and 3) decreased the variability observed for these land use types, assessed as the range between low and high mean estimates. However, the confidence for the typical values provided for the aggregated annual and permanent crop categories in block 1 is relatively high compared to the confidence in estimates for categories of blocks 2 and 3 (Supporting Information A, Figure S6).

The few crops identified at the beginning of the assessment as misclassified were corrected as oilseed crops and legumes in block 2 and clover seed in block 3. Most of the abundance estimates for these categories showed a high consensus, with the sole exception of low-abundance estimates for oilseed crops. However, given that the estimates for these categories were the result of only one round of assessment, the resulting CFs are presented for illustrative purposes and are not recommended as readily applicable without further assessment.

### 3.2 Generic CFs for Potential Land Use Impacts on Pollinator Abundance

**Figure 2:**
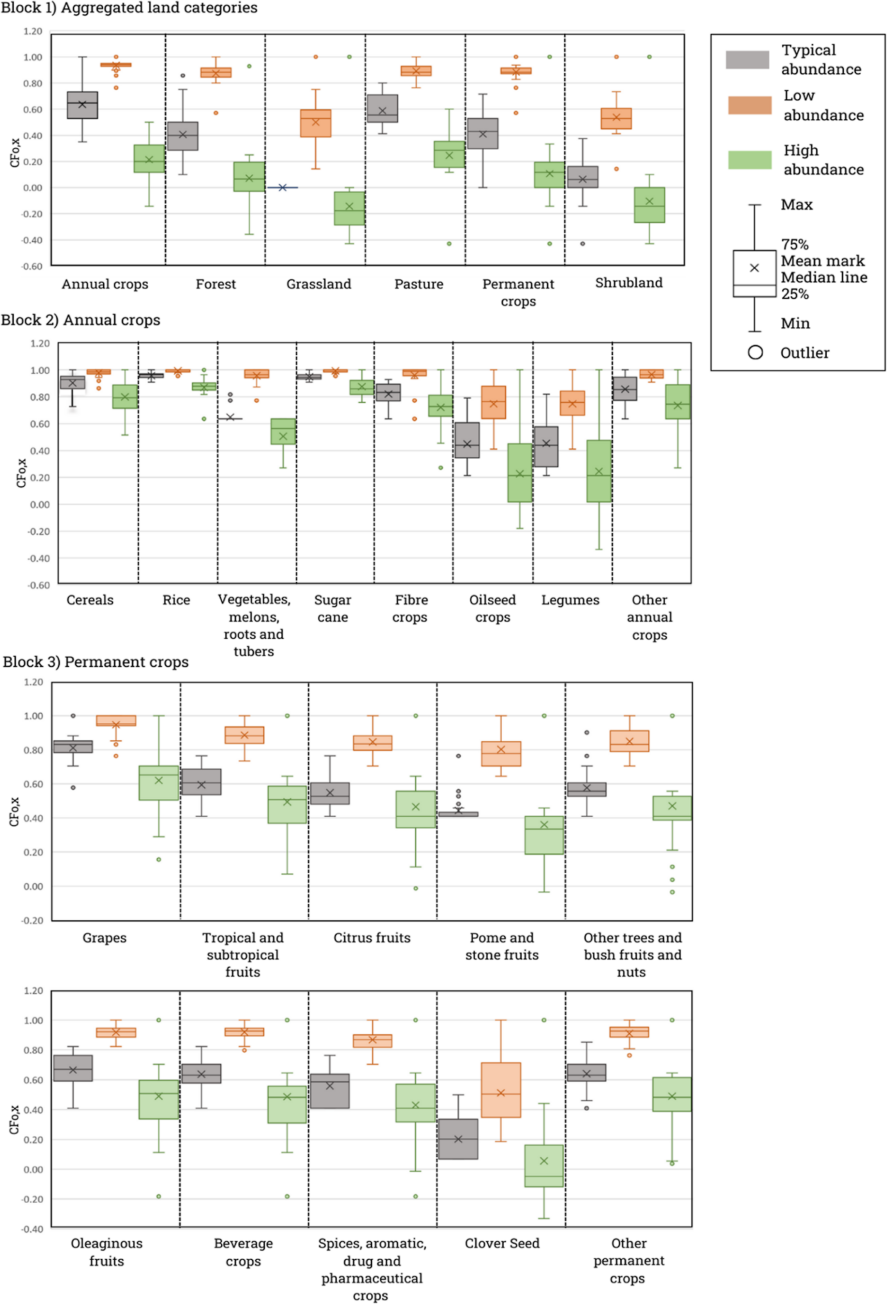
CFs for land occupation impacts on pollinator abundance (m^2^·year/m^2^·year reference land).

The pollinator abundance estimates from each expert were used to derive CFs for land occupation impacts, as described in section 2.1. The resulting CFs (CF_O,*x*_) are shown in Figure 2 (full table of CFs can be seen in Supporting Information A, Table S1, along with combined and weighted uncertainty for each land use category and further specification on CFs derived from estimates that did not reach consensus). The CFs are described as “dimensionless” as they represent a given number of pollinators relative to the maximum abundance of a reference land (m^2^·year/m^2^·year reference land since land occupation flows are commonly expressed in LCA with the unit m^2^·year).

**Figure 3:**
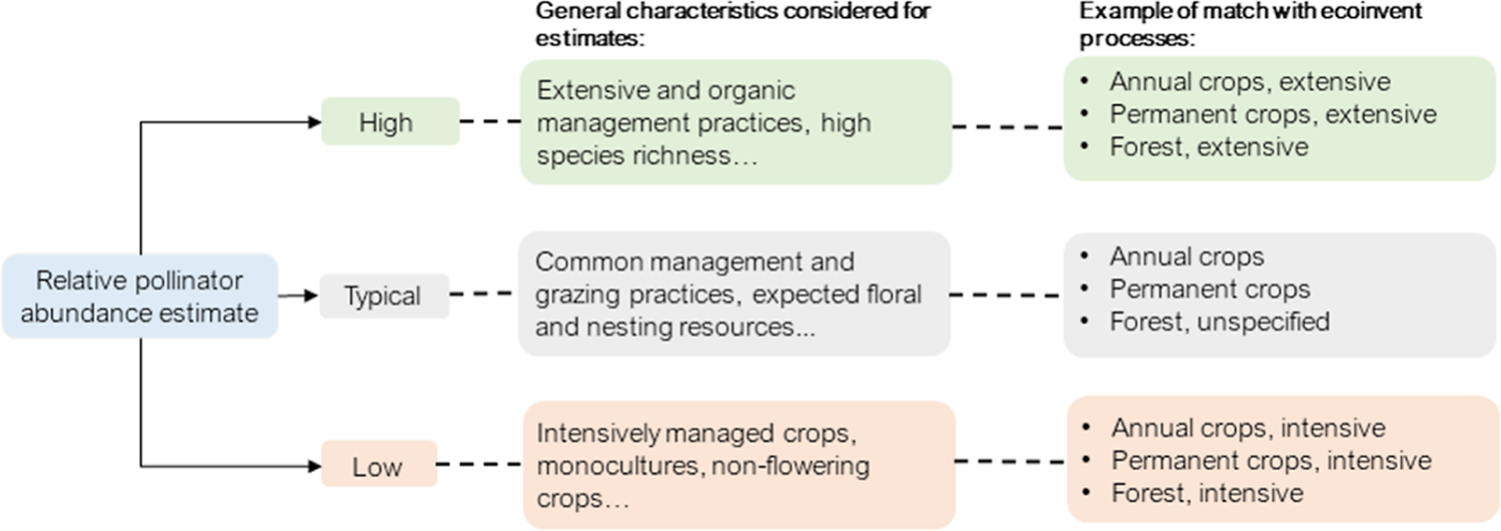
Considerations by experts for pollinator abundance estimates and their compatibility with land use intensity levels found in the ecoinvent inventory.

Experts provided short written argumentations describing their considerations for each level of abundance along with their quantitative estimates. The main characteristics associated with low-abundance estimates were non-flowering landscapes, which present low foraging and nesting resources as well as intensive, high chemical input, monoculture practices. High pollinator abundance estimates were generally associated with extensive management practices, low to no chemical input, rich understory, and rich flowering plants. Given the detailed considerations made for each level of abundance and consistency in descriptions between experts, we recommend applying the low-abundance CFs to elementary flows that specify intensive practices and the high-abundance CFs to elementary flows that describe extensive management practices. This aligns with recent efforts^38^ to provide guidance on the application of CFs and avoid arbitrary selection that can lead to deviating results. The CFs for typical estimates can be applied to generic flows where locations and management practices are unspecified (Figure 3). After normalization in reference to grassland, estimates of high pollinator abundance above 100 resulted in negative CFs, reflecting positive impacts to pollinator abundance, which can be associated with land presenting exceptionally high quality of foraging and nesting resources or under active restoration and maintenance practices. An indication of uncertainty for each CF is provided by a measure of dispersion, assessed, in this case, as the SD.

## 4 Discussion

### 4.1 Considerations of Expert Elicitation Assessment to Characterize Pollinator Abundance

The use of Delphi assessment for the derivation of comparable pollinator abundance estimates resulted in a comprehensive set of scores based on careful considerations from the experts involved in the assessment. This assessment allowed for the quantification of the potential impact on the relative pollinator abundance associated with diverse land use categories. Generally, the development of CFs requires simplifications and compromises to match the information available in life cycle inventories with the modeling of complex human–environment dynamics. In this case, the relationship between land use and pollinator relative abundance was assessed with the use of estimates based on expert knowledge and derived through a Delphi expert elicitation method. The Delphi assessment allowed us to quantify the relative differences in pollinator abundance associated with 24 land use categories, providing valuable data in terms of not only quantifiable estimates for characterization but also recommendations that can be used for improvements of LCA databases and considerations in future studies.

The feedback provided by multiple experts, whose expertise combined covers an ample geographical scope, showed that their estimates were based on careful considerations regarding conventional practices and management of major crop types as well as on variations that could emerge from seasonal and geographical differences. According to the argumentation submitted by the experts along with their scores, the type of management practices was one of the most influential factors for the variability of abundance not only within but also between crops. This reiterates the need to incorporate more detail regarding management practices at an elementary flow level by expanding the application of keywords such as “intensive” and “extensive” flows to most agricultural flows.

The relative pollinator abundance scores and thus CFs are consistent with trends observed in recent years regarding pollinator abundance. For example, annual crops, which are usually intensively managed, were linked in several studies to the lowest expected abundance and richness of pollinator communities,^8,9^ while natural grasslands were commonly found to harbor the highest abundance rates,^6^ helping increase species richness in comparison with annual crops.^8^ However, it is important to notice that the method proposed in this study is based on averaged relative values and may not always be comparable to results from local measurements or predictions performed in a site-specific area.^42^ Moreover, the high divergence observed for multiple low-abundance estimates may highlight the need for further field and on-site research to verify the state of pollinator communities and allow for a better comparison of relative differences. While no confidence scores were provided for low and high estimates, the consistently high divergence of scores for low-abundance estimates could indicate intrinsic regional and management variations or a general lack of certainty and knowledge regarding the extent of pollinator abundance decrease in poor-quality areas and intensively managed landscapes.

### 4.2 Dealing with Uncertainty

When dealing with data derived from expert elicitation methods, there are generally three main sources of uncertainty. These are generally described as within-expert uncertainty, between-experts uncertainty, and the uncertainty that can be attributed to the data itself (e.g., due to real heterogeneity,^42^ misclassifications, etc.).^41,43^ Within-expert uncertainty occurs when an expert is unsure about the state or assessed quality of a particular land category (described as well as imperfect knowledge). To minimize within-expert uncertainty, participants were asked to submit their scores for up to three rounds and were encouraged to review the summary feedback. Additionally, experts provided a score of their confidence level for typical abundance estimates, which was used to interpret the variation in typical scores across rounds.

Between-experts uncertainty arises from disagreement among experts. The disagreements can be due to differences in, for example, expertise, heterogeneity of the land classifications, or cognitive biases.^43^ To decrease between-experts uncertainty, the Delphi method relies on consecutive rounds of scoring where experts provide argumentation for their estimates, which can then be considered by the other experts during their re-evaluation of scores. To decrease the risk of forced consensus that can arise from group dynamics, the participants were kept anonymous during the assessment, and everyone provided the survey results independently. The variation and convergence levels were assessed at the end of each round. As pointed out by the panel of experts, there were a handful of crops that were misclassified. These crops were separated into new categories and reassessed in the third round of the Delphi assessment.

To quantify the associated uncertainty of the pollinator abundance estimates produced in this study, we used a measure of dispersion, the SD. The CFs were produced for each land category and are presented along with their SD as well as combined and weighted measures of uncertainty. Future studies could focus on the potential use of uncertainty measures to assess the global sensitivity of the CFs and move toward regionalization of impacts to better reflect biogeographical differences.^44^

### 4.3 Application in LCA and Recommendations

The CFs for aggregated land categories assessed in block 1 are directly applicable to the current elementary flow list of ecoinvent. To exemplify their application, we include a brief illustrative comparison of two hypothetical agricultural products (Supporting Information C), detailing the relevant inventory analysis and characterization of each product to assess the associated pollinator abundance decrease. The CFs for the more specific land use categories assessed in blocks 2 and 3 can be selected based on unit processes within an inventory database.

While this study focused on the development of world-generic CFs for occupation impacts, pollinator communities and their capacity to provide pollination services are influenced by a range of biogeographical characteristics and agricultural land-use intensities that vary across the globe.^45^ To address these differences, country-specific CFs could be derived in future studies by matching the land use categories assessed in this study with land cover maps and/or land system archetypes to produce regionalized CFs that can represent the potential impact of occupying land in a given country or spatial unit chosen.^46,47^ Furthermore, the geographies considered by the expert panel on their estimations of pollinator abundance cover 45 countries (see Supporting Information A, Figure S1) from across all continents and representative biomes. However, additional input from experts on regions such as North and South Africa, as well as East Asia, could be the target of future efforts to improve the representativeness of the CFs.

While the derivation of CFs for transformation impacts were beyond the scope of this study, their assessment is essential to account for the impacts of land cover change.^48^ However, current discrepancies in the operationalization of transformation impact assessment should be addressed in order to improve the compatibility of new CFs with inventory LCA flows and improve the accuracy of the assessment. From a pragmatic point of view, it would be recommendable and effective to provide CFs addressing a net transformation impact that can be directly linked to a single inventory flow (e.g., “from annual to permanent crops”) instead of adjusting to the current structure where transformation flows are separated as two separate flows (“from” and “to”).^38^ The midpoint indicator result can be linked in future research to endpoint categories. For example, “ecosystem quality” could reflect the relation between decreased pollinator abundance and potential decrease in plant species richness, while “human health” could reflect malnutrition damages through agricultural productivity losses.

The inputs provided by experts indicate that protective land practices such as the maintenance or restoration of hedgerows and flower rich field margins can have a considerable influence on the expected pollinator abundance, even in crop areas where intensive management practices take place.^49,50^ CFs for active restoration or enhancement activities can be included as negative CFs to represent their potential improvement on the expected pollinator abundance and allow for their consideration in the selection of land use practices when comparing among product systems. This is of significant value to support decision and policy making where analyses are made not only during design stages for the prevention of impacts but also to compare among remediation strategies where restoration measures are needed. Moreover, the high SD in some of the land use categories assessed indicates the need to increase the level of detail provided in the elementary flows, as was the case for the category of forest. Given the general consensus, dense, coniferous, monotypic, or intensively managed forests will likely support limited pollinator abundance in comparison with open, deciduous, and tropical forests with understory vegetation. The inclusion of a few relevant keywords, such as the aforementioned, would better allow the differences within this category to be reflected.

The results of this study provide evidence of the applicability of expert elicitation methods to fill gaps where quantitative information might be missing from available sources for interdisciplinary applications such as impact assessment methods. This was further exemplified with the proven application of the resulting CFs in a hypothetical comparison between two crops, where key differences were observed on the pollinator abundance decline associated with each alternative. While the degree of pollinator abundance is of high relevance for its associated capacity to provide the ecosystem with the service of pollination, multiple other aspects remain as well of high concern, such as pollinator diversity and persistence of rare species. Future research could target the characterization of such additional environmental impacts as well as the continuous improvement of the CFs produced in this study with the aim of providing representative results that can aid in preventing further declines of wild pollinators.

## Supporting Information Available

Geographical distribution of expert panel and areas of expertise; boxplots for normalized *S*_*x*_ estimates of block 1; boxplots for normalized *S*_*x*_ estimates of block 2; convergence of *S*_*x*_ expert scores; boxplots for normalized *S*_*x*_ estimates of block 3; confidence of experts on typical scores of abundances; and CFs for land occupation impacts on pollinator abundance (PDF)
Normalized *S*_*x*_ estimates of pollinator abundance (XLSX)
Example of the CFs’ application (XLSX)
