# Supplementary material for: Characterization Factors to Assess Land Use Impacts on Pollinator Abundance in Life Cycle Assessment
Source: Environ Sci Technol. 2023 Feb 13;57(8):3445–54. doi: 10.1021/acs.est.2c05311 (PMC9979645; doi:10.1021/acs.est.2c05311)
Supplement: Supplementary file 1 — es2c05311_si_001.pdf [file es2c05311_si_001.pdf]

Supporting Information: A

Manuscript: Characterization factors to assess land use impacts on pollinator abundance in Life Cycle Assessment

Journal: Environmental Science & Technology

Authors: Elizabeth M. Alejandre[1,2*], Laura Scherer[1], Jeroen B. Guinée[1], Marcelo A. Aizen[3], Matthias Albrecht[4], Mario V. Balzan[5], Ignasi Bartomeus[6], Danilo Bevk[7], Laura A. Burkle[8], Yann Clough[9], Lorna J. Cole[10], Casey M. Delphia[11], Lynn V. Dicks[12,13], Michael P.D. Garratt[14], David Kleijn[15], Anikó Kovács-Hostyánszki[16], Yael Mandelik[17], Robert J. Paxton[18,19], Theodora Petanidou[20], Simon Potts[14], Miklós Sárospataki[21], Catharina J.E. Schulp[22], Menelaos Stavrinides[23], Katharina Stein[24], Jane C. Stout[25], Hajnalka Szentgyörgyi[26], Androulla I. Varnava[23], Ben A. Woodcock[27], Peter M. van Bodegom[1]

Number of pages: 9

Content list:

Figure S1. Geographical distribution of expert panel and areas of expertise (Page S2)

Figure S2. Boxplots for normalized *Sx* estimates of Block 1(Page S3)

Figure S3. Boxplots for normalized *Sx* estimates of Block 2 (Page S4)

Figure S4. Convergence of S_x expert scores (Page S5)

Figure S5. Boxplots for normalized *Sx* estimates of Block 3 (Page S6)

Figure S6. Confidence of experts on typical scores of abundances (Page S7)

Table S1. Characterization factors for land occupation impacts on pollinator abundance (Page S8-S9)

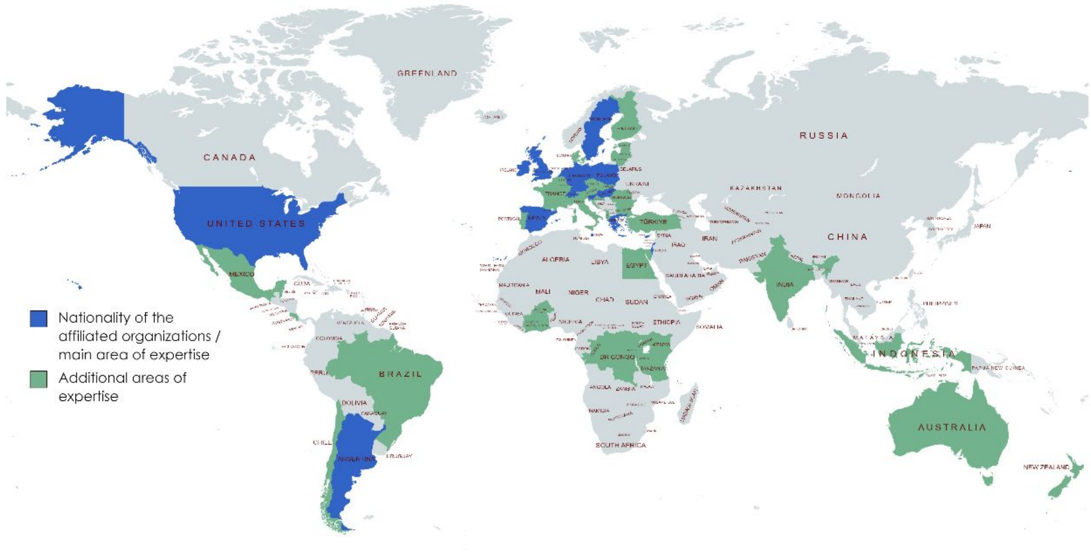

Figure S1. Geographical distribution of expert panel covering 16 affiliated nationalities, and areas of expertise covering 45 countries. Created with Mapchart.net.

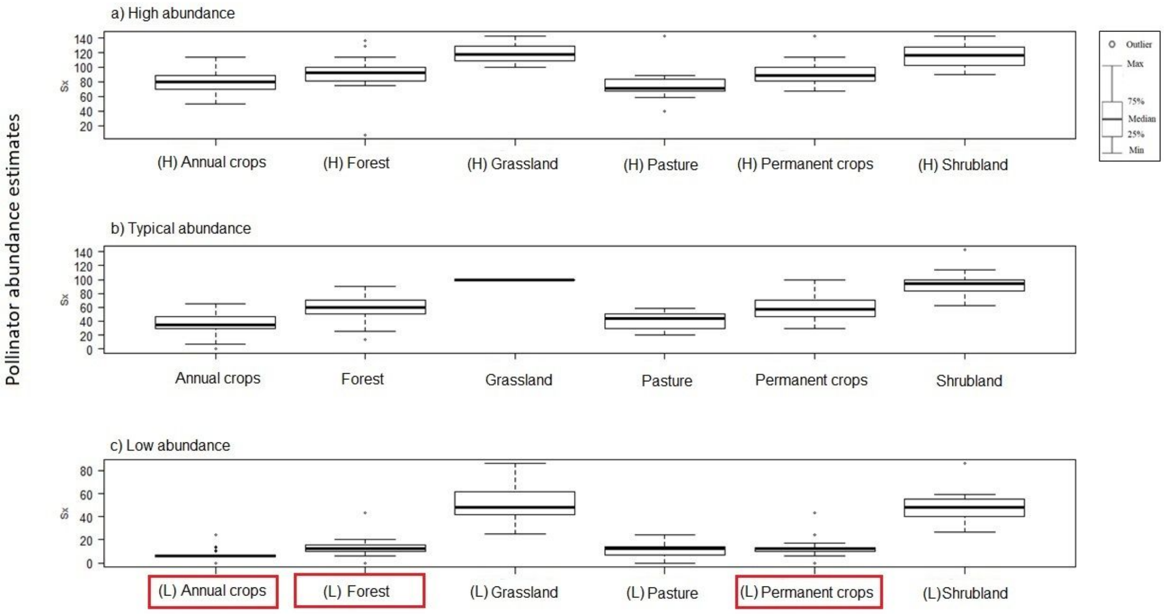

Figure S2. Boxplots for normalized pollinator abundance estimates (high, typical, and low) of aggregated land categories from Block 1. The categories that did not reach consensus are in red boxes. Note y-axes are at different scales.

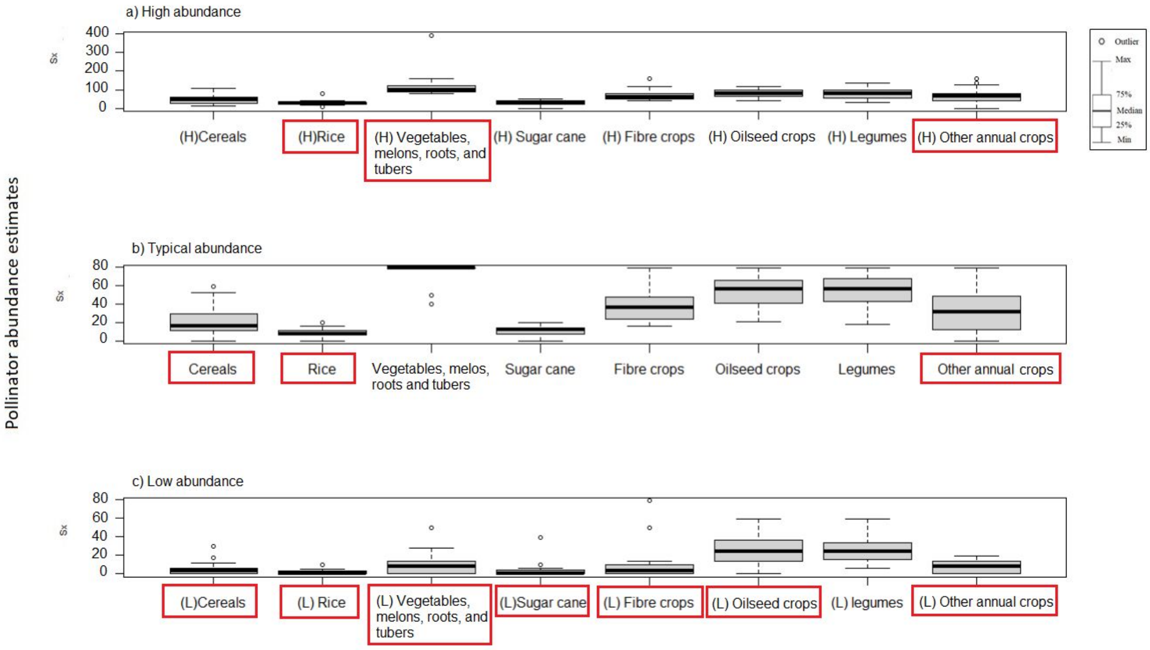

Figure S3. Boxplots for normalized pollinator abundance estimates (high, typical and low) of Annual crops categories from Block 2. Land categories that did not reach consensus are in red boxes. Note y-axes are at different scales.

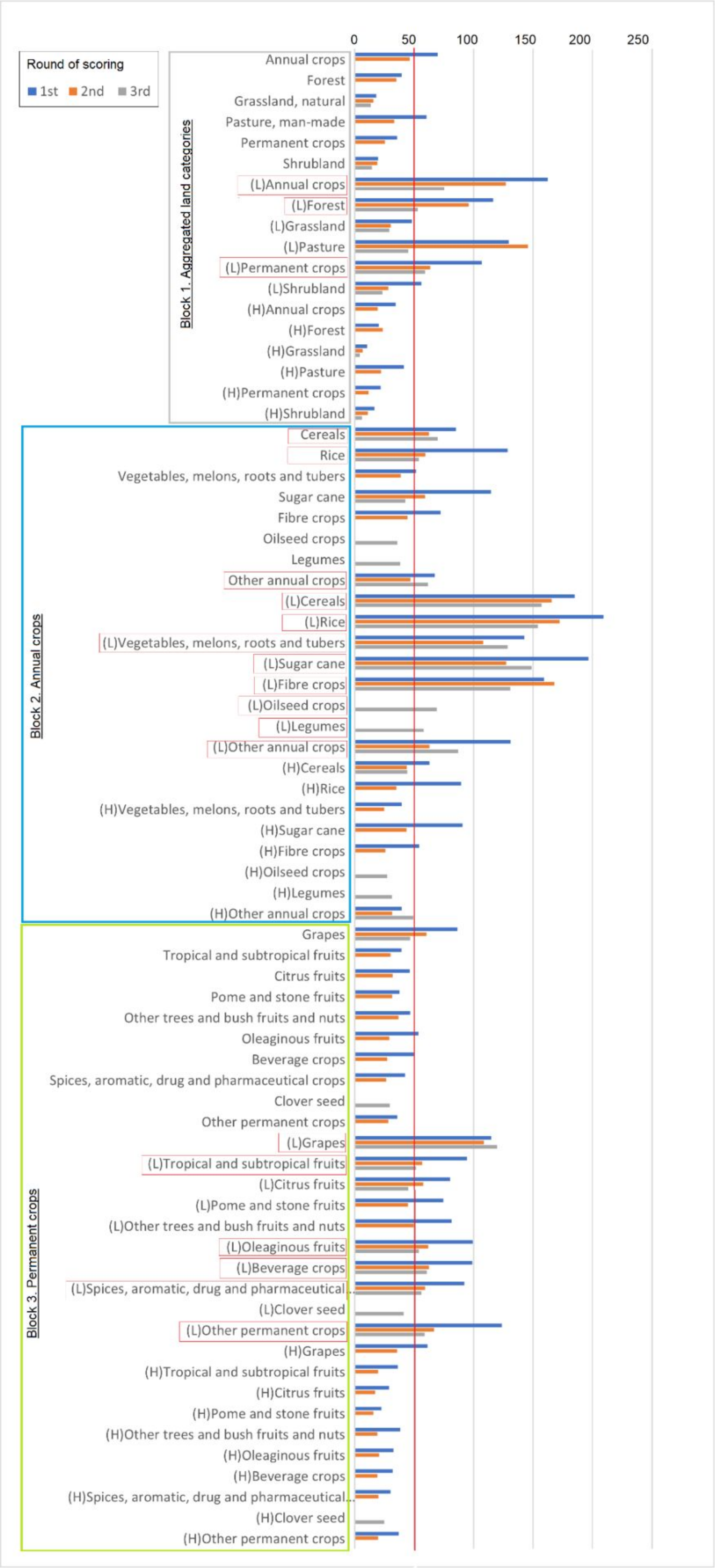

Figure S4. Coefficient of variation for pollinator abundance estimates across three rounds of surveying all land use categories and levels (typical, low and high). Values of ≤50 were considered to have reached consensus. Land categories that did not reach consensus are in red boxes.

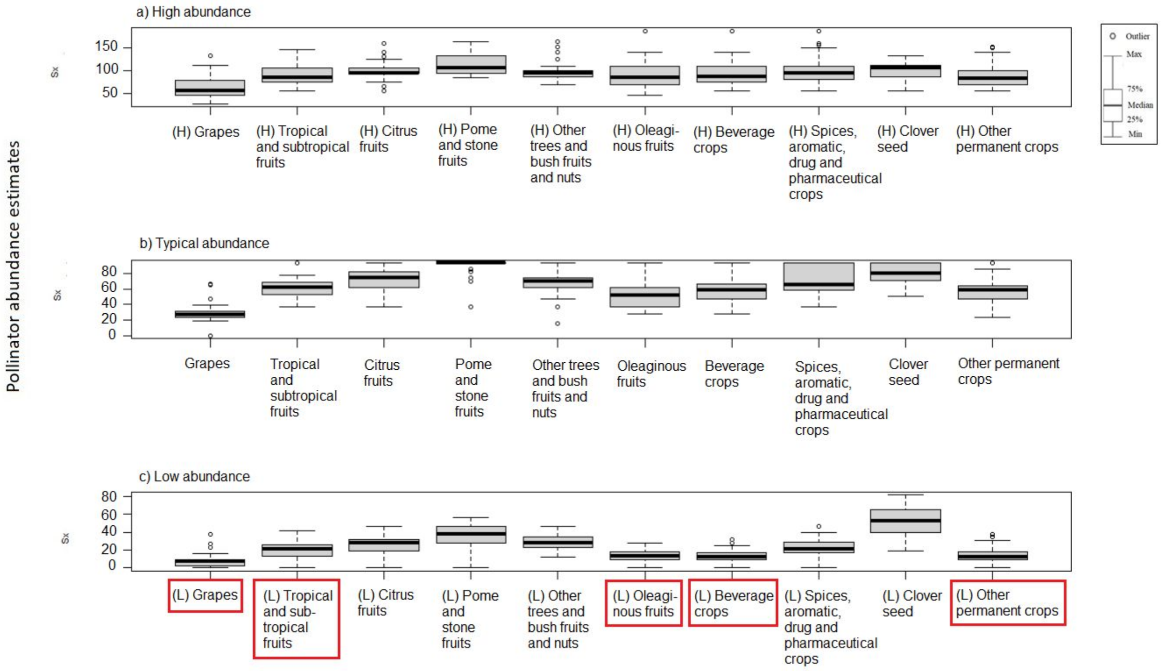

Figure S5. Boxplots for normalized pollinator abundance estimates (high, typical, and low) of permanent crop categories from Block 3. Land categories that did not reach consensus are in red boxes. Note y-axes are at different scales.

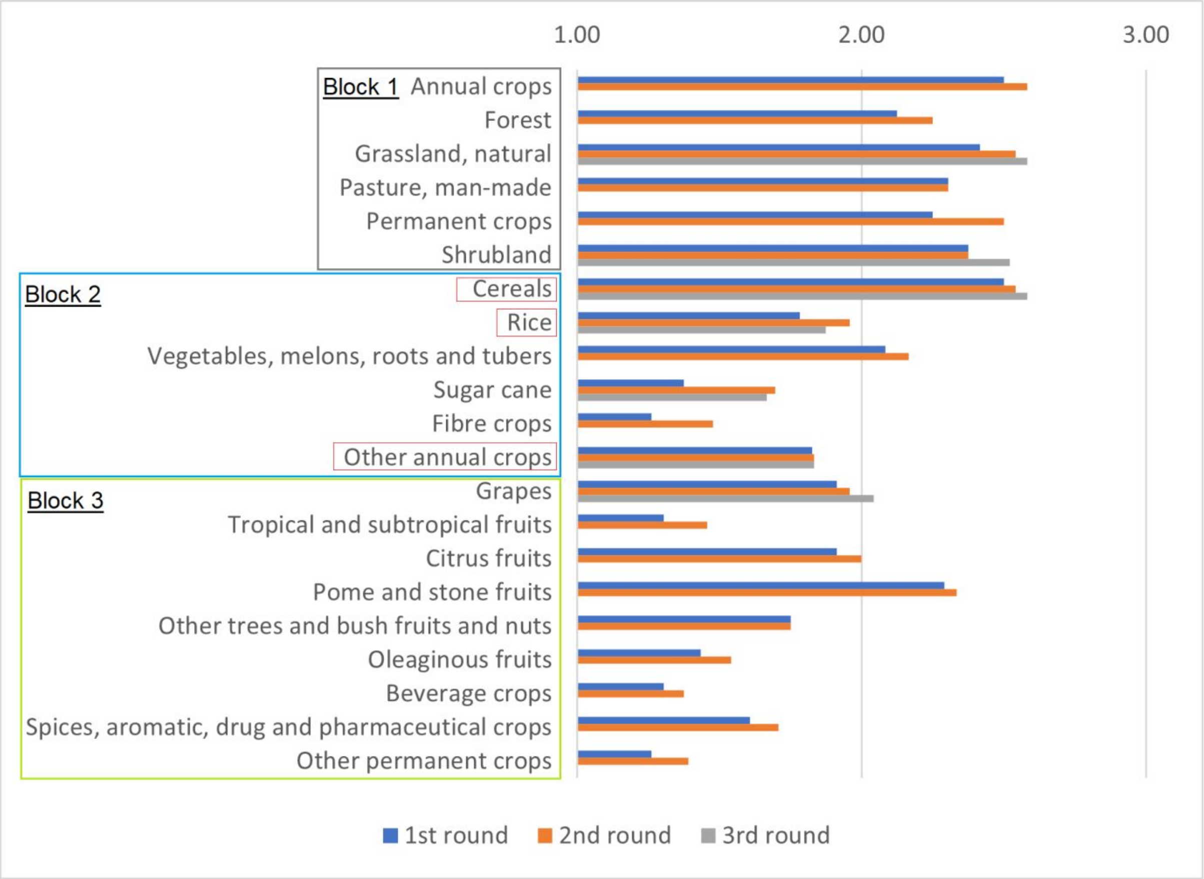

Figure S6. Average confidence scores ranging from 1-3 (1=Low, 2=Moderate, 3=High) for typical pollinator abundance estimates across three rounds of surveying. Those scores that reached consensus after two rounds of surveys did not undergo a third round. Land categories that did not reach consensus after 3 rounds are in red boxes.

Table 1. Characterization factors for land occupation impacts on pollinator abundance ($m^2 \cdot year/ m^2 \cdot year$ reference land). CFs derived from estimates that did not reach a consensus are shown in red and italic format. * Results based on one round of scoring.

	Typical		Low		High			
**Block 1: Aggregated land use categories**	$CF_{o,x}$	SD of $CF_{o,x}$	$CF_{o,x}$	SD of $CF_{o,x}$	$CF_{o,x}$	SD of $CF_{o,x}$	Combined uncertainty	Weighted uncertainty
Annual crops	0.64	0.16	*0.93*	0.05	0.21	0.16	0.33	0.29
Forest	0.41	0.19	*0.87*	0.08	0.07	0.25	0.38	0.34
Grassland	0	0.00	0.48	0.18	-0.19	0.27	0.33	0.29
Pasture	0.59	0.11	0.89	0.05	0.25	0.18	0.29	0.26
Permanent crops	0.41	0.16	*0.88*	0.08	0.07	0.24	0.37	0.33
Shrubland	0.06	0.16	0.52	0.15	-0.15	0.28	0.34	0.31
**Block 2: Annual crops**	$CF_{o,x}$	SD of $CF_{o,x}$	$CF_{o,x}$	SD of $CF_{o,x}$	$CF_{o,x}$	SD of $CF_{o,x}$	Combined uncertainty	Weighted uncertainty
Cereals	*0.79*	0.14	*0.95*	0.07	0.53	0.24	0.24	0.22
Rice	*0.91*	0.05	*0.98*	0.02	*0.69*	0.17	0.16	0.14
Vegetables, melons, roots and tubers	0.24	0.10	*0.89*	0.11	*-0.16*	0.65	0.57	0.49
Sugar cane	0.89	0.05	*0.97*	0.08	0.71	0.13	0.14	0.13
Fibre crops	0.61	0.18	*0.90*	0.19	0.32	0.29	0.32	0.29
Oilseed crops*	0.45	0.17	*0.75*	0.17	0.23	0.28	0.30	0.27
Legumes*	0.45	0.18	0.75	0.18	0.25	0.30	0.30	0.27
Other annual crops	*0.69*	0.22	*0.92*	0.07	*0.35*	0.41	0.35	0.32
**Block 3: Permanent crops**	$CF_{o,x}$	SD of $CF_{o,x}$	$CF_{o,x}$	SD of $CF_{o,x}$	$CF_{o,x}$	SD of $CF_{o,x}$	Combined uncertainty	Weighted uncertainty
Grapes	0.70	0.14	*0.91*	0.09	0.35	0.27	0.29	0.26
Tropical and subtropical fruits	0.36	0.18	*0.81*	0.11	0.10	0.26	0.35	0.32
Citrus fruits	0.29	0.15	0.75	0.13	0.00	0.24	0.35	0.31
Pome and stone fruits	0.12	0.13	0.65	0.12	-0.14	0.24	0.37	0.32

Other trees and bush fruits and nuts	0.33	0.16	0.72	0.09	0.01	0.25	0.33	0.29
Oleaginous fruits	0.47	0.16	*0.87*	0.08	0.09	0.32	0.38	0.33
Beverage crops	0.43	0.15	*0.86*	0.09	0.04	0.31	0.38	0.34
Spices, aromatic, drug and pharmaceutical crops	0.31	0.18	0.78	0.12	-0.02	0.35	0.40	0.36
Clover seed*	0.20	0.14	0.51	0.21	0.06	0.28	0.29	0.26
Other permanent crops	0.43	0.15	*0.85*	0.11	0.09	0.30	0.37	0.33
